# Targeted therapeutics for pancreatitis

**DOI:** 10.3389/fphys.2026.1795419

**Published:** 2026-03-13

**Authors:** Tareq Alsaleh, John George

**Affiliations:** 1 Department of Internal Medicine, AdventHealth Orlando, Orlando, FL, United States; 2 Department of Gastroenterology and Hepatology, AdventHealth Orlando, Orlando, FL, United States; 3 Pancreas Center, AdventHealth Orlando, Orlando, FL, United States

**Keywords:** acute pancreatitis, chronic pancreatitis, pancreatitis, recurrent acute pancreatitis, targeted therapeutics

## Abstract

**Importance:**

Acute pancreatitis (AP) can result in significant morbidity and mortality. Its complications include persistent organ failure, necrosis, and death. Recurrent episodes of AP may also result in chronic pancreatitis (CP), a fibroinflammatory condition characterized by chronic pain and endocrine and exocrine failure. Despite improved supportive care, approved disease-modifying targeted therapies for AP or CP are lacking.

**Observations:**

Mechanistic studies identify early pathways within acinar and ductal cells that lead to injury, providing potential therapeutic targets before necrosis and other complications develop. Sustained cytosolic calcium elevation drives premature enzyme activation and mitochondrial failure, maintained by store-operated calcium entry through Orai1. Calcium overload promotes increased mitochondrial permeability, ATP depletion, and necrotic cell death. Potential early interventions include Orai1 inhibition (CM4620/Auxora with early-phase human safety data and phase 2b signals), mitochondrial pore inhibition (NIM811) and other mitochondrial protectants. When systemic inflammation escalates, preclinical containment targets include NLRP3 inflammasome signaling, neutrophil extracellular traps, damage-associated molecular pattern signaling (including HMGB1), and upstream cytokine shedding via ADAM17/TACE. Pragmatic strategies under study, such as early high-energy feeding, test whether modifiable supportive inputs can shift early severity trajectories. In chronic pancreatitis, long-term disease modification centers on targeting pancreatic stellate cells, which are critical drivers of fibrosis. Furthermore, the highly morbid chronic pain of CP can be modified through treatment of neuroimmune pain sensitization. The emerging clinical pipeline includes repurposed anti-fibrotic or immunomodulatory agents (pirfenidone, paricalcitol, tocilizumab, proglumide), genotype-matched therapy (CFTR modulation in selected populations), and cell-based approaches.

**Conclusion and Relevance:**

The current targeted therapeutic landscape in pancreatitis is promising, but difficulties lie in trial enrichment as the timing of drug administration is critically dependent on the timeline of disease. Therefore, progress will depend on matching treatment timing to target biology, identifying patients using practical early severity and inflammatory features, and using endpoints that reflect disease modification (organ failure, necrosis, recurrence, fibrosis progression, pancreatic function, and pain trajectories). Trial designs matching these therapeutics to their target biology will help understand the real clinical value of mechanism-based regimens across the AP–RAP–CP continuum.

## 1 Introduction

Pancreatitis is a clinical spectrum characterized by pancreatic inflammation and tissue injury. It may present as acute pancreatitis (AP), recurrent acute pancreatitis (RAP), and chronic pancreatitis (CP). Acute pancreatitis, an acute inflammatory process of the pancreas, is diagnosed when two of the following criteria are met: characteristic abdominal pain, serum amylase or lipase at least three times the upper limit of normal, and imaging findings consistent with AP. Recurrent acute pancreatitis is diagnosed when two distinct episodes of AP occur with complete resolution between episodes. Over time, recurrent attacks may progress to CP, a fibroinflammatory syndrome characterized by irreversible structural change, chronic pain, and exocrine and endocrine dysfunction (Mederos et al., 2021).

Acute pancreatitis can lead to organ failure, pancreatic necrosis and infected necrosis, intensive care unit admission, prolonged hospitalization, and death. Mortality ranges from 1% to 5% overall but exceeds 30% in severe necrotizing disease. In contrast, CP commonly drives long-term morbidity through chronic pain, frequent hospitalization, opioid exposure, exocrine pancreatic insufficiency, and type 3c diabetes (Mederos et al., 2021).

Clinically, AP unfolds in overlapping phases with multiple therapeutic windows. The early phase is dominated by acinar and ductal cell injury and rapid immune activation that can progress to systemic inflammatory response syndrome (SIRS) and organ failure. A later phase involves local complications, including necrosis and infection, and ongoing systemic complications. These distinct phases in pathogenesis support a staged strategy that prioritizes early cellular rescue, followed by selective containment of maladaptive innate immune responses, and then longer-term interventions that target remodeling and chronicity.

Current management of pancreatitis remains largely supportive. In AP, this includes fluid resuscitation, analgesia, nutritional support, and complication management. In CP, treatment is mainly symptomatic and includes pain control, endoscopic or surgical interventions, and enzyme and nutritional support. Despite improvements in supportive care and interventions, there are no approved disease-modifying pharmacologic or biologic therapies for AP or CP. A major barrier is heterogeneity across etiologies and difficulty in predicting severity (Phillips et al., 2024).

## 2 Pathogenic mechanisms with clinically actionable targets

**FIGURE 1:**
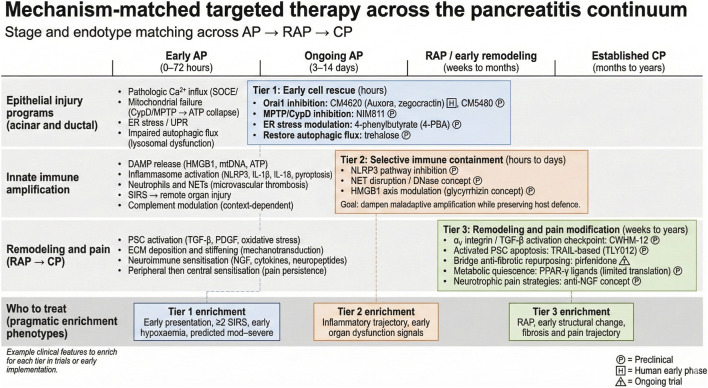
Mechanism-matched targeted therapy across the pancreatitis continuum. Schematic linking dominant biological processes across acute pancreatitis (AP), recurrent acute pancreatitis (RAP), and chronic pancreatitis (CP) to therapeutic windows and representative targeted interventions. The timeline highlights early AP (0–72 h), ongoing AP (3–14 days), RAP/early remodeling (weeks to months), and established CP (months to years). Tier 1 strategies prioritize early epithelial rescue by limiting pathologic calcium influx, preserving mitochondrial energetics, reducing ER stress, and restoring autophagic flux. Tier 2 strategies aim to selectively contain maladaptive innate immune amplification, including DAMP signaling, NLRP3 activation, and neutrophil extracellular trap (NET)-mediated injury, to reduce systemic inflammation while preserving host defense. Tier 3 strategies target longer-horizon remodeling and pain biology, including pancreatic stellate cell activation, extracellular matrix remodeling, and neuroimmune sensitization. Boxes at the bottom provide pragmatic enrichment phenotypes that may help select patients most likely to benefit from each tier in trials or early implementation. Evidence badges indicate development stage (P, preclinical; H, human early phase; T, clinical trial ongoing).

In the pancreas, acute injury begins within acinar and ductal cells, then rapidly recruits innate immune amplification. With recurrent or persistent injury, remodeling processes dominate, including fibrogenesis and neuroimmune sensitization. Rather than managing these as independent, isolated pathways, a more practical view is that severe disease is often a result of a small number of coupled pathways: sustained calcium entry with ATP collapse, failure of cellular quality-control systems, and escalating innate immune injury. These mechanisms recur across various etiologies, providing tractable targets (Figure 1).

### 2.1 Early epithelial injury: calcium overload, mitochondrial failure, and cellular quality-control breakdown

Calcium ions are tightly regulated intra- and extracellularly. In healthy acinar cells, Ca^2+^ signaling is compartmentalized and occurs as brief, repetitive apical Ca^2+^ oscillations triggered by physiologic secretagogues. These localized signals support stimulus–secretion coupling, including regulated zymogen granule exocytosis, while limiting spread to the basal cytosol and nucleus. Mitochondria near the apical pole help match ATP production to secretory demand and buffer local Ca^2+^ transients, supporting ATP-dependent Ca^2+^ clearance and cellular homeostasis (Petersen et al., 2021).

In pancreatitis, this pattern shifts from localized oscillations to sustained cytosolic Ca^2+^ elevation. Sustained Ca^2+^ overload promotes premature trypsinogen activation and early acinar dysfunction. Pathogenic stimuli, including bile acids, fatty acid ethyl esters, and sustained cholecystokinin hyperstimulation, can trigger this transition (Petersen et al., 2021; Pallagi et al., 2020). Sustained cytosolic Ca^2+^ elevation also promotes ER Ca^2+^ release and pathological Ca^2+^ entry via store-operated calcium entry channels, particularly Orai1 (Wen et al., 2015). In addition to the ER, acinar cells contain acidic Ca^2+^ stores in endolysosomal compartments concentrated in the apical region. Under physiologic conditions, Ca^2+^ release from these stores contributes to apical signaling and can interact with ER Ca^2+^ dynamics. In pancreatitis-relevant settings, abnormal acidic-store Ca^2+^ release and impaired refilling can promote Ca^2+^ spread and sustain cytosolic Ca^2+^ elevation, linking lysosomal dysfunction to Ca^2+^-driven injury programs (Petersen et al., 2011).

Mitochondrial injury is both a consequence and amplifier of Ca^2+^ toxicity. Ca^2+^ overload promotes opening of the mitochondrial permeability transition pore (MPTP), leading to ATP depletion, reactive oxygen species generation, and necrotic cell death (Maléth and Hegyi, 2016; Xia et al., 2025). Cyclophilin D is a key mediator of MPTP opening through Ca^2+^-dependent and Ca^2+^-independent pathways. The Ca^2+^-independent mechanism includes direct inhibition of ATP synthase, which is relevant in L-arginine–induced severe AP (Biczo et al., 2018). ATP depletion then limits Ca^2+^ extrusion, reinforcing Ca^2+^ overload and mitochondrial failure, creating a vicious cycle.

In parallel, stressed acinar cells rely on cellular quality-control systems to maintain homeostasis. Pathogenic stress can overwhelm the ER, causing unfolded protein accumulation and activation of the unfolded protein response (UPR). The UPR reduces folding burden, increases folding capacity, and enhances clearance of misfolded proteins. When these adaptive responses fail, signaling can shift toward apoptosis and inflammatory amplification (Biczo et al., 2018; Read and Schröder, 2021). ER stress also interacts bidirectionally with Ca^2+^ dysregulation and mitochondrial dysfunction in AP.

Autophagy is another core quality-control mechanism that preserves homeostasis by clearing damaged organelles and protein aggregates (Biczo et al., 2018). In AP, impaired autophagic flux promotes abnormal zymogen activation and necrotic injury (Zhang et al., 2023). This dysfunction can reflect impaired cathepsin processing, reduced autophagosome–lysosome fusion (e.g., reduced syntaxin 17), and impaired lysosomal biogenesis (e.g., TFEB degradation) (Mareninova et al., 2009; Wang et al., 2023; Wang et al., 2019). Restoring autophagic flux with trehalose reduced trypsinogen activation and necrosis in experimental AP (Biczo et al., 2018). Because lysosomes also participate in Ca^2+^ signaling, lysosomal dysfunction can couple impaired autophagy with disordered Ca^2+^ handling, amplifying mitochondrial stress and necrotic injury (Pascua-Maestro et al., 2017).

### 2.2 Innate immune amplification and microvascular injury

Acinar and ductal injury releases damage-associated molecular patterns (DAMPs), including HMGB1, ATP, mitochondrial DNA, and histones. These ligands activate pattern recognition pathways on immune cells and propagate inflammation. Cytokine and chemokine networks amplify rapidly, with IL-1β, IL-6, TNF-α, and CXCL1 contributing to local inflammation and SIRS (Venkatesh et al., 2022; Glaubitz et al., 2023). IL-6 correlates with severity and remains a practical biomarker candidate with therapeutic relevance (Chen et al., 2016; Chao et al., 2006). An important intracellular sensor, NLRP3, detects these inflammatory signals and drives inflammasome assembly in pancreatic cells, which further contributes to immune amplification. This promotes release of IL-1β and IL-18 and supports gasdermin D–mediated pyroptosis. These pathways contribute to pancreatitis severity and systemic amplification (Swanson et al., 2019; Liu et al., 2023).

Neutrophils are a major effector cell type in severe AP. Neutrophil infiltration and neutrophil extracellular trap formation contribute to severe AP by promoting tissue injury and microvascular thrombosis and may worsen remote organ injury. In addition, complement activation can modulate inflammation and vascular injury, with context-dependent effects that may amplify or shape disease severity (Venkatesh et al., 2022; Antkowiak et al., 2022).

### 2.3 Transition to chronicity: fibrogenesis and pain neurobiology

With recurrent or persistent injury, remodeling processes dominate. Activation of pancreatic stellate cells (PSCs) is a central driver of pancreatic fibrogenesis. Cytokines, oxidative stress, growth factors such as TGF-β and PDGF, and paracrine signals from injured epithelium convert quiescent PSCs into activated myofibroblast-like cells. Activated PSCs proliferate and secrete extracellular matrix proteins, particularly type I collagen (Antkowiak et al., 2022; Mews et al., 2002). Collagen deposition is followed by cross-linking and matrix stiffening. These changes reinforce mechano-transduction pathways that sustain PSC activation (Mews et al., 2002). TGF-β and SMAD signaling remains a dominant profibrotic axis and includes modulators such as Hic-5 that may provide therapeutic leverage (Gao et al., 2020).

Chronic inflammation also drives structural and functional changes in pancreatic innervation. Increased nerve density, nerve hypertrophy, and perineural inflammation correlate with pain severity (Demir et al., 2015). Neurotrophic factors, especially nerve growth factor, promote nerve fiber growth (Olesen et al., 2017). Inflammatory mediators lower nociceptor activation thresholds and drive peripheral sensitization. Persistent nociceptive input can then remodel spinal cord and brain pain processing and cause central sensitization, hyperalgesia, and allodynia (Frøkjær et al., 2012).

## 3 Discussion

### 3.1 Targeted therapeutics for acute pancreatitis

Therapeutic development in AP is most compelling when tied to time-sensitive biology and actionable clinical contexts. A recurring theme is that early epithelial rescue targets are most plausible when delivered very early, whereas immune-directed strategies require careful selection and safety monitoring to avoid unintended harm.

#### 3.1.1 Intra-acinar targets

Orai1 is the pore-forming subunit of store-operated calcium entry channels and mediates sustained pathological calcium influx after ER calcium depletion by AP triggers. First-generation inhibitors such as GSK-7975A and CM_128 reduced calcium overload, trypsinogen activation, and necrosis in mouse and human acinar cells with high inhibition in experimental systems (Wen et al., 2015). However, limited isoform selectivity constrained their translational potential. GSK-7975A inhibited Orai1 and Orai2 with similar potency and partially inhibited Orai3 (Zhang et al., 2020). This raised concern for unintended effects given the distinct physiologic roles across Orai isoforms. Its efficacy was also affected by pore geometry and selectivity filter configuration, suggesting an allosteric mechanism rather than direct pore blockade (Derler et al., 2013). Further, GSK-7975A demonstrated activity against vascular smooth muscle and non-selective cation channels, raising concerns for hemodynamic adverse effects (De Moudt et al., 2021). Newer generation Orai1 inhibitors, including CM4620 (auxora) and CM5480 aimed to address these limitations.

CM4620 demonstrates higher functional activity in cells expressing Orai1. This includes pancreatic acinar cells and immune cells, where it reduces neutrophil oxidative burst and cytokine production. It also modulates PSC biology, suggesting a broader impact on fibroinflammatory pathways (Waldron et al., 2019). In a phase 2 open-label dose-response study in AP with SIRS and hypoxemia, short-term safety appeared acceptable and serious adverse events did not increase relative to standard care. Potential benefit was reported across clinical recovery endpoints, including reduced persistent SIRS, improved tolerance of solid food, and shorter hospitalization (Charles et al., 2021). These findings supported continued evaluation in randomized and blinded studies. In CARPO (phase 2b), Auxora was evaluated in patients with AP and accompanying SIRS. Publicly reported results have described faster clinical recovery and fewer respiratory and necrotizing complications in treated groups compared with placebo (Sutton et al., 2024). Although these findings require validation in fully peer-reviewed reporting and subsequent trials, they support the concept that early calcium pathway containment can translate into clinical recovery benefits when utilized in the right clinical contexts.

CM5480 has been proposed as a more selective Orai1 inhibitor with potential relevance to ductal dysfunction. Preclinical work suggests protection against bile acid and ethanol related ductal impairment and restoration of epithelial secretory function (Pallagi et al., 2022).

CM5480 has also been proposed as a candidate that could influence the AP to RAP to CP continuum by reducing sustained calcium dysregulation and downstream fibroinflammatory responses in preclinical models (Szabó et al., 2023). At present, its translational status in pancreatitis remains defined primarily by preclinical evidence, and published human trial data are not yet available.

A key limitation of Orai1 inhibition is timing. The therapeutic window appears narrow in experimental systems, with greatest benefit when administered within 1–6 h of AP onset (Wen et al., 2015; Waldron et al., 2019). In clinical practice, delayed presentation and heterogeneity across etiologies may reduce their impact. Longer-term safety data are also needed, particularly regarding infection risk and immune function given their immunomodulatory effects (Charles et al., 2021).

The MPTP is a convergent effector pathway in AP. Early calcium overload and oxidative stress activate cyclophilin D and trigger MPTP opening. This leads to mitochondrial membrane potential collapse, ATP depletion, and necrotic cell death (Maléth and Hegyi, 2016). NIM811 (N-methyl-4-isoleucine cyclosporin) is a non-immunosuppressive cyclosporin A derivative that inhibits cyclophilin D. In preclinical AP models, it restored mitochondrial membrane potential, preserved mitochondrial mass, and reduced injury across multiple triggers including bile acids, ethanol, and fatty acids. Protective effects were seen in ductal and acinar cells. *In vivo*, NIM811 reduced edema, necrosis, leukocyte infiltration, and serum amylase across cerulein, ethanol fatty acid, and taurocholic acid models (Tóth et al., 2019). It also preserved bicarbonate transport in ductal cells, which may support barrier function against premature enzyme activation. Its oral bioavailability and lack of immunosuppression make it a good candidate for clinical trial, but early timing of drug administration and appropriate patient selection remain critical.

Lipid peroxidation is a downstream consequence of mitochondrial dysfunction and contributes to membrane injury. Aldehyde dehydrogenase 2 plays an important role in reducing lipid peroxidation by degrading toxic lipid aldehydes. Encouragingly, Alda-1 is an aldehyde dehydrogenase 2 activator that reduced pancreatic enzymes, lipid peroxidation products, and apoptotic markers in cerulein induced AP in mice (Li et al., 2022).

High temperature requirement protein A2 (HtrA2) is a mitochondrial serine protease linked to mitochondrial homeostasis. It is crucial for maintaining cellular protein balance and triggering apoptosis by regulating caspases when cells are exposed to stress. Deoxyarbutin has been described as acting on the HtrA2 and PGC-1α axis and restoring mitochondrial membrane potential, ATP production, and autophagy. *In vitro* work demonstrated reduced necrosis and reactive oxygen species accumulation in primary acinar cells (Cao et al., 2020).

Despite promising preclinical data, human antioxidant trials have shown mixed results. High-dose or combination supplementation has shown no benefit and potential harm in some studies. Key limitations include suboptimal dosing, route, and timing, as well as altered pharmacodynamics in acute illness (Coman et al., 2025). Future approaches require targeted formulations, early administration strategies, and enriched patient selection.

4-phenylbutyrate is a chemical chaperone that reduces ER stress by stabilizing protein conformation and supporting proper folding. In caerulein plus lipopolysaccharide and L-arginine models, 4-phenylbutyrate reduced AP severity and downregulated ER stress markers, including GRP78, CHOP, and phospho-eIF2α. It also reduced necroptosis pathway markers, including RIP3 and phospho-MLKL, *in vitro* and *in vivo* (Han et al., 2022).

Trehalose enhanced autophagic efficiency and restored autophagic flux in experimental AP. In L-arginine induced AP, trehalose reduced trypsinogen activation, necrosis, and pancreatic injury markers (Biczo et al., 2018; Abu-El-Haija et al., 2018). Proposed mechanisms include improved autophagosome to lysosome fusion and enhanced lysosomal degradative capacity. This supports a strategy that prioritizes completion of autophagy rather than simply increasing autophagosome formation.

#### 3.1.2 Immune-containment targets

Immune-directed strategies are most plausible when positioned as selective containment of maladaptive amplification rather than broad suppression. This approach aims to reduce SIRS and organ injury while preserving host defense and tissue repair (Sendler et al., 2020).

NLRP3 inflammasome activation contributes to inflammatory amplification in severe AP. Pharmacologic NLRP3 inhibition has reduced disease severity and inflammatory cytokine production in experimental AP and has been linked to reduced immune cell infiltration and tissue injury in mechanistic models (Sendler et al., 2020). Neutrophil extracellular traps contribute to pancreatic injury, microvascular thrombosis, and organ dysfunction in severe disease. Experimental work supports neutrophil extracellular trap disruption as a strategy to reduce pancreatic inflammation and trypsin activation in severe AP models. These findings motivate exploration of neutrophil extracellular trap-directed approaches as adjunctive containment strategies in enriched severe inflammatory phenotypes (Merza et al., 2015). HMGB1 is a DAMP released during pancreatic injury and contributes to sustained inflammatory signaling. Experimental HMGB1 inhibition strategies, including glycyrrhizin in animal models, have shown reductions in inflammatory mediators and pancreatic injury severity. This supports DAMP pathway targeting as a mechanistically aligned containment strategy, particularly when systemic inflammation becomes self-sustaining (Yasuda et al., 2006; Sawa et al., 2006; Pan, 2014).

ADAM17 (also known as TNF-α converting enzyme, TACE) regulates ectodomain shedding of inflammatory mediators, including TNF-α and the IL-6 receptor. In experimental acute pancreatitis, the ADAM17–IL-6 axis appears to amplify neutrophil recruitment and pancreatic tissue injury. Targeting this pathway reduced pancreatic inflammation and attenuated downstream organ injury signals in mechanistic models, supporting ADAM17 inhibition as another “upstream” containment strategy in hyperinflammatory phenotypes. However, ADAM17 has broad physiologic roles in epithelial repair and host defense, so safety, timing, and careful patient selection will be central to translation into clinical practice (Saad et al., 2022).

#### 3.1.3 Pragmatic and supportive targets

Magnesium is a physiological calcium antagonist and may influence calcium-dependent injury pathways that precede trypsin activation and mitochondrial stress. Furthermore, hypomagnesemia is common in critical illness and has been associated with worse outcomes in severe acute pancreatitis cohorts, raising interest in magnesium status as a potentially modifiable factor. Currently, clinical evidence for magnesium as a treatment in established acute pancreatitis is limited, and much of the interventional human literature relates to prevention settings (for example, post-procedure pancreatitis) rather than treatment of established disease. A practical framing is to emphasize early detection and repletion of documented deficiency while acknowledging the need for dedicated trials that test dosing, timing, and target populations (Pergolini et al., 2024). Consistent with this biologic rationale, an RCT of 270 patients showed a protective effect of magnesium supplementation against post-ERCP pancreatitis (Aletaha et al., 2022).

Early nutrition may affect the course of acute pancreatitis by limiting catabolism and supporting gut barrier function. A randomized study from Hungary tested whether giving patients more calories early (about 30 kcal/kg/day) improve outcomes compared with a lower-calorie strategy during the early phase of acute pancreatitis. Although this is not a drug therapy, it highlights an important point: some early, modifiable interventions can be tested using outcomes that matter clinically, such as progression to severe disease and development of complications (Márta et al., 2017).

### 3.2 Targeted therapeutics for chronic pancreatitis

Disease modification in CP is most likely to emerge from interventions that target PSC activation, matrix remodeling, and neuroimmune sensitization. These mechanisms are interdependent and converge on fibrosis, pain trajectories, and functional decline.

#### 3.2.1 Pancreatic stellate cell activation: The central target

TGF-β is a dominant regulator of fibrogenesis and induces PSC activation and collagen synthesis through SMAD-dependent signaling (Singh et al., 2019). αV integrins participate in extracellular activation of latent TGF-β1 and represent an upstream checkpoint. CWHM-12, an αV integrin inhibitor, reduced pancreatic fibrosis development in mice and has been reported to reverse established fibrosis in preclinical models without reducing acute injury severity (Uc et al., 2016). These findings support anti-fibrogenic specificity and emphasize reversal as a meaningful preclinical benchmark for therapies intended for established early CP.

TLY012 is a soluble trimeric form of human tumor necrosis factor related apoptosis-inducing ligand (TRAIL). It has been developed to target activated myofibroblasts and activated PSCs while sparing quiescent PSCs. In preclinical CP models, TRAIL-based strategies have been described as reducing fibrosis and pancreatic injury. This approach is notable because it also targets fibrosis-associated pain phenotypes in animal models, supporting a dual anti-fibrotic and analgesic intent (Phillips et al., 2024). The translational appeal includes mechanism-based cellular selectivity and potential synergy with broader strategies that reduce ongoing inflammatory drive.

Pirfenidone is approved for idiopathic pulmonary fibrosis and is being repurposed as a candidate disease-modifying therapy in predicted moderately severe and severe AP with the goal of reducing progression along the RAP to CP trajectory. Preclinical work suggests anti-inflammatory and anti-fibrotic effects, including modulation of cytokine balance.

A pilot clinical trial has been initiated to evaluate safety and tolerability in patients with predicted moderately severe and severe AP and includes exploratory clinical endpoints (Phillips et al., 2024). If proven safe in acutely ill patients, pirfenidone could serve as a bridge strategy during high-risk acute episodes to reduce cumulative fibroinflammatory injury. Peroxisome proliferator-activated receptor gamma ligands, including troglitazone, have also demonstrated anti-fibrotic effects in experimental CP by promoting PSC quiescence and reducing collagen synthesis. Clinical translation has been constrained by safety limitations of specific agents in this class (Shimizu, 2008; Talukdar and Tandon, 2008).

#### 3.2.2 Neuroimmune remodeling and pain sensitization

Immune and neuronal crosstalk drives chronic pain in CP. Cytokines sensitize nociceptors, while activated neurons release neuropeptides that further activate immune cells and reinforce a feed-forward loop. Therapeutic strategies include cytokine inhibition, neuropeptide receptor antagonism, and neurotrophic factor inhibition, including anti-nerve growth factor approaches developed for other chronic pain syndromes (Glaubitz et al., 2023). These approaches remain largely preclinical in CP and are most compelling when paired with interventions that reduce peripheral drivers such as inflammation and fibrosis.

## 4 Other therapies in the translational pipeline

Several other candidate interventions are entering early clinical evaluation (Table 1). These generally fall into two categories: repurposed immunomodulatory or anti-fibrotic agents being tested in defined CP or high-risk AP phenotypes, and etiology-matched approaches that target genetically mediated or channelopathy-driven disease.

**TABLE 1:** Selected ongoing or recently completed clinical trials evaluating emerging therapies and supportive interventions across acute and chronic pancreatitis.

Therapy/trial	Primary mechanism	Intended setting	Trial status/phase
Auxora (CM4620; zegocractin)	Orai1/CRAC calcium-entry inhibition	AP with SIRS	Phase 2b completed (CARPO trial). (Waldron et al., 2019)
Pirfenidone	Anti-fibrotic and immunomodulatory	Predicted moderately severe/severe AP (progression prevention concept)	Active clinical trial listed in trial registries. Phase 2; recruiting. (Bava et al., 2025)
High-energy feeding (GOULASH)	Early nutritional strategy (higher caloric delivery)	Early AP	Protocol published; trial designed to compare high vs. lower energy. (Márta et al., 2017)
Paricalcitol (VDR agonist)	Anti-fibrotic and immune-modulatory signaling	CP	Pilot clinical trial in recruitment phase. (Pandol, 2025)
Tocilizumab (TOPAC)	IL-6 pathway inhibition	Painful CP	Recruiting. (Hagn-Meincke et al., 2025)
Proglumide (CCK receptor antagonist)	Reduces CCK-mediated stimulation; downstream inflammation/fibrosis hypotheses	CP	Phase 1 human trial published. (Ciofoaia et al., 2024)
Ivacaftor (CFTR potentiator)	CFTR potentiation (improves ductal ion/fluid secretion)	CFTR-related pancreatitis/recurrence reduction	Active, not recruiting. (Mission, 2025)
Autologous MSC and islet co-transplantation	Regenerative/metabolic (T3c)	CP patients undergoing TP-IAT	Recruiting. (Wang, 2025)
Naldemedine	Peripheral µ-opioid receptor antagonism (mechanism explored for RAP prevention)	RAP prevention	Phase 2 (registry + protocol published). (Cook et al., 2023)

Abbreviations: AP, acute pancreatitis; CP, chronic pancreatitis; RAP, recurrent acute pancreatitis; SIRS, systemic inflammatory response syndrome; CRAC, calcium release–activated calcium; VDR, vitamin D receptor; IL-6, interleukin-6; CCK, cholecystokinin; CFTR, cystic fibrosis transmembrane conductance regulator; MSC, mesenchymal stromal cells; TP-IAT, total pancreatectomy with islet autotransplantation; T3c, pancreatogenic diabetes (type 3c). Trial status reflects published reports and trial registries at the time of writing.

### 4.1 Vitamin D receptor (VDR) agonism

Paricalcitol is under evaluation in a pilot CP trial to test feasibility and symptom control, consistent with a remodeling-directed rationale that may be relevant in fibrotic and pain phenotypes (Pandol, 2025).

### 4.2 IL-6 pathway modulation

Tocilizumab is being studied in CP as a targeted immune-modulatory strategy. This approach is conceptually aligned with selective containment in patients with evidence of immune-driven symptom biology, but it requires careful safety monitoring given infection risk with IL-6 blockade (Olesen, 2024).

### 4.3 CCK receptor antagonism

Proglumide is being evaluated in early-phase CP studies focused on tolerability and feasibility. This strategy targets neurohormonal stimulation biology that may contribute to pain and inflammatory flares in selected patients (Ciofoaia et al., 2024).

### 4.4 Statins

Simvastatin has been tested as a prevention strategy for recurrent acute pancreatitis. However, a recent randomized trial has reported no meaningful reduction in recurrence, which supports positioning statins as an evidence-defined “repurposing” approach rather than a default disease-modifying strategy (Guilabert et al., 2026).

### 4.5 CFTR modulation

Ivacaftor and other CFTR modulators have been associated with reduced pancreatitis events in CFTR gating mutation populations, supporting a precision approach in pancreatitis related to cystic fibrosis (Carrion et al., 2018).

### 4.6 Cell-based and regenerative strategies

Mesenchymal stromal cell (MSC) programs are being studied in CP, including trials focused on pain outcomes and peri-transplant islet support in TP-IAT populations. These approaches fit best as adjunctive “repair” strategies that target pain biology or metabolic endpoints rather than core injury containment (Wang, 2022; Wang et al., 2018).

## 5 Conclusion

Pancreatitis is a dynamic continuum in which early epithelial injury, immune amplification, and progressive remodeling interact over time. Disease-modifying therapy will require matching the timing of treatment to the dominant biology and selecting the patients most likely to respond, rather than treating “acute” or “chronic” pancreatitis as uniform labels.

A practical way to organize targeted therapy is a staged framework. Early strategies aim to stabilize acinar and ductal cell injury by limiting pathologic calcium influx, preserving mitochondrial energetics, reducing maladaptive stress responses, and restoring autophagic flux. Intermediate strategies aim to blunt harmful innate immune amplification by targeting DAMP signaling, inflammasome activation, and neutrophil-driven injury when systemic inflammation begins to dominate outcomes. Later strategies focus on remodeling and pain biology by targeting stellate cell activation, extracellular matrix remodeling, and neuroimmune sensitization that drive fibrosis, pain, and functional decline.

This approach also clarifies patient selection. Clinically useful selection features include early physiologic severity, signs of systemic inflammatory escalation, and markers of evolving structural disease. Combining these features with etiology and disease stage can help align patients with mechanism-matched interventions.

Several recurring issues have slowed progress in pancreatitis trials. Many epithelial rescue strategies likely require treatment within a short window after symptom onset, so delayed enrollment can dilute benefit. “Acute pancreatitis” also groups biologically distinct entities with different trajectories, making all-comer designs prone to mixing responders and non-responders. Endpoints are often misaligned with the target and time course of effect, and immune-directed approaches must be designed with safety-first monitoring and prespecified discontinuation rules. Future trials should treat time-to-treatment, mechanism-matched inclusion criteria, and biology-aligned endpoints as core design variables. Platform and combination-ready designs may be especially useful because they can support staged regimens that mirror the clinical course.

Overall, pancreatitis care is moving from describing mechanisms to testing therapies that target those mechanisms in selected patients. Progress will depend on intervening early enough to matter, choosing targets that match the dominant biology in each case, and using outcomes that reflect true disease modification rather than short-term symptom relief.
